# Robot-assisted laparoscopic colpectomy in female-to-male transgender patients; technique and outcomes of a prospective cohort study

**DOI:** 10.1007/s00464-016-5333-8

**Published:** 2016-11-14

**Authors:** Freek Groenman, Charlotte Nikkels, Judith Huirne, Mick van Trotsenburg, Hans Trum

**Affiliations:** 1 Center of Expertise on Gender Dysphoria, VU University Medical Center, Boelelaan 1117, 1081 HV Amsterdam, The Netherlands; 2 Department of Obstetrics and Gynecology, VU University Medical Center, Amsterdam, The Netherlands

**Keywords:** Robot, Laparoscopy, Hysterectomy, Colpectomy, Transgender

## Abstract

**Background:**

Gender-affirming surgeries in female-to-male (FtM) transgender patients include mostly hysterectomy, bilateral salpingo-oophorectomy and mastectomy. Sometimes further surgery is performed, such as phalloplasty. Colpectomy may be performed to overcome gender dysphoria and disturbing vaginal discharge; furthermore, it may be important in reducing the risk of fistulas due to the phalloplasty procedure with urethral elongation. Colpectomy prior to the reconstruction of the neourethra seems to reduce fistula rates on the very first anastomosis. Therefore, at our center, colpectomy has become a standard procedure prior to phalloplasty and metoidioplasty with urethral elongation. Colpectomy is known as a procedure with potentially serious complications, e.g., extensive bloodloss, vesicovaginal fistula or rectovaginal fistula. Colpectomy performed via the vaginal route can be a challenging procedure due to lack of exposure of the surgical field, as many patients are virginal. Therefore, we investigated whether robot-assisted laparoscopic hysterectomy with bilateral salpingo-oophorectomy (TLH–BSO) followed by robot-assisted laparoscopic colpectomy (RaLC) is an alternative for the vaginal approach.

**Methods:**

Robot TLH/BSO and RaLC as a single-step procedure was performed in 36 FtM patients in a prospective cohort study.

**Results:**

Median length of the procedure was 230 min (197–278), which reduced in the second half of the patients, median blood loss was 75 mL (30–200), and median discharge was 3 days (2–3) postoperatively. One patient with a major complication (postoperative bleeding with readmission and transfusion) was reported.

**Conclusion:**

To our knowledge, this is the first report of RaLC. Our results show that RaLC combined with robot TLH–BSO is feasible as a single-step surgical procedure in FtM transgender surgery. Future studies are needed to compare this technique to the two-step surgical approach and on its outcome and complication rates of subsequent phalloplasty.

**Electronic supplementary material:**

The online version of this article (doi:10.1007/s00464-016-5333-8) contains supplementary material, which is available to authorized users.

Gender dysphoria is a condition in which people suffer from incongruence between their natal sex and their gender identity, i.e., their experienced gender [1].

Individuals with gender dysphoria differ in the extent to which they physically desire to transition to the opposite sex. The majority of patients will receive cross-sex hormone treatment. Many female-to-male (FtM) patients request mastectomy, hysterectomy and bilateral salpingo-oophorectomy (BSO). A subcutaneous mastectomy is performed, and a male appearance of the thorax is created [2, 3]. Mastectomy can be combined with hysterectomy and BSO. Although the majority of FtM patients have the wish to undergo further genital surgery, for different reasons only few have further surgery, e.g., phalloplasty.

At this stage, only few transmen clearly express an actual wish for phalloplasty with urethral lengthening. More often transmen ask for colpectomy (removal of vaginal epithelium) sometime after having had mastectomy and hysterectomy/BSO. Vaginal discharge in general and/or increased lubrication as a consequence of sexual arousal is often experienced negatively and can interfere with the male self-esteem. Simple closure of the introitus is no option as vaginal outflow obstruction may cause accumulation of secretions and hydrocolpos. This may result in a wish to have a colpectomy performed. Besides these indications, colpectomy is gaining in importance preparatory to phalloplasty with urethral elongation. The anastomosis of the origin ostium urethrae and the reconstructed tube functioning as extended urethra is known to be very prone to fistulas. Studies on urethral lengthening in phalloplasty surgery reported 16 to 68% on urethral fistula [4–7]. The incidence of urethrocutaneous fistula in patients undergoing phalloplasty without colpectomy were 27% [5] and 30% [7], and when a colpectomy was performed, the incidence of fistula was 16% [6] and 68% [4]. Schaff et al. described that the majority of the developed fistulas were originated at the connection site of the lengthened urethra to the prelaminated urethra and not due to the colpectomy. Occurrence of urethral fistula after metoidioplasty surgery, including colpectomy, was reported by Perovic et al. [9], Takamatsu et al. [10] and Djordjevic et al. [8] to be 7.7, 13.6 and 17.4%, respectively. It is hypothesized—but not yet confirmed—that colpectomy and consecutive obliteration of the vagina may reduce tensile forces at the neourethral junction. Therefore, at our center, colpectomy has become a prerequisite for phalloplasty with urethral lengthening.

Colpectomy is often described as a challenging and complicated operation [11]. As a result of a previously performed hysterectomy, the bladder may be overlying the vaginal apex and also the rectovaginal septum may contain scar tissue due to previous surgery. The resulting alteration in anatomy poses an elevated risk of bladder, ureteral or rectal injury. Vesicovaginal, rectovaginal and ureterovaginal fistula, and intestinal lesions may occur [12]. These complications induce severe morbidity and require additional surgery. Although these complications are rare, they can have a major impact on a patient’s quality of life. Other intra-operative risks consist of blood loss due to the extensive blood supply to and from the vaginal wall (arterial supply: vaginal, uterine, internal pudendal and middle rectal arteries; venous supply: vaginal venous plexus draining into internal iliac vessels). The blood supply is possibly enhanced under influence of supraphysiological cross-sex hormonal treatment. Postoperative bleeding can lead to pelvic floor or retroperitoneal hematomas. Other frequently encountered problems are transient bladder voiding difficulties requiring intermittent catheterization or indwelling urinary catheter for a longer period [13].

An additional difficulty in the vaginal approach may be the access to the vagina. Many patients are nulliparous and virginal. Furthermore, patients are under continuous cross-sex hormonal treatment, resulting in a narrow vagina with considerable atrophy of the epithelium [14, 15] leading to poor exposure of the operating area [14, 15].

In this paper, we describe a novel technique in which a robot-assisted laparoscopic hysterectomy (robot TLH) and BSO is combined with a robot-assisted laparoscopic colpectomy (RaLC) as a single-step procedure. This technique may prove to be beneficial over the vaginal colpectomy and laparoscopic hysterectomy as two different procedures. Besides the advantage of combining two procedures, RaLC potentially has less complication risk and shorter hospital stay than the vaginal approach. Although there have been reports of laparoscopic colpectomy with low complication rate, they describe that in many cases, part of the vagina still needs to be removed vaginally [16]. We chose the robot over conventional laparoscopy because of a potential larger learning curve with conventional laparoscopic colpectomy, the need for vaginal removal of remnant vagina, 3D high-definition camera for fine surgery using the robot, low intra-abdominal pressure possibility using the robot and experience with robotic surgery in our center.

## Materials and methods

### Setting

We performed a single-center prospective cohort study in 36 female-to-male transgender patients. Patients underwent the procedure as described below after informed consent was obtained. The study protocol (2016.224) has been approved by the Medical Ethics Review Committee of VU University Medical Centre (OHRP number IRB00002991). The FWA number assigned to VU University Medical Center is FWA00017598.

Patients were included between July 2011 and June 2015. One gynecologist performed all procedures with extensive experience in robotic surgery including robot-assisted hysterectomies and oncological surgery (JT).

### Robot-assisted laparoscopic hysterectomy with bilateral salpingo-oophorectomy and colpectomy in four steps

**Fig. 1:**
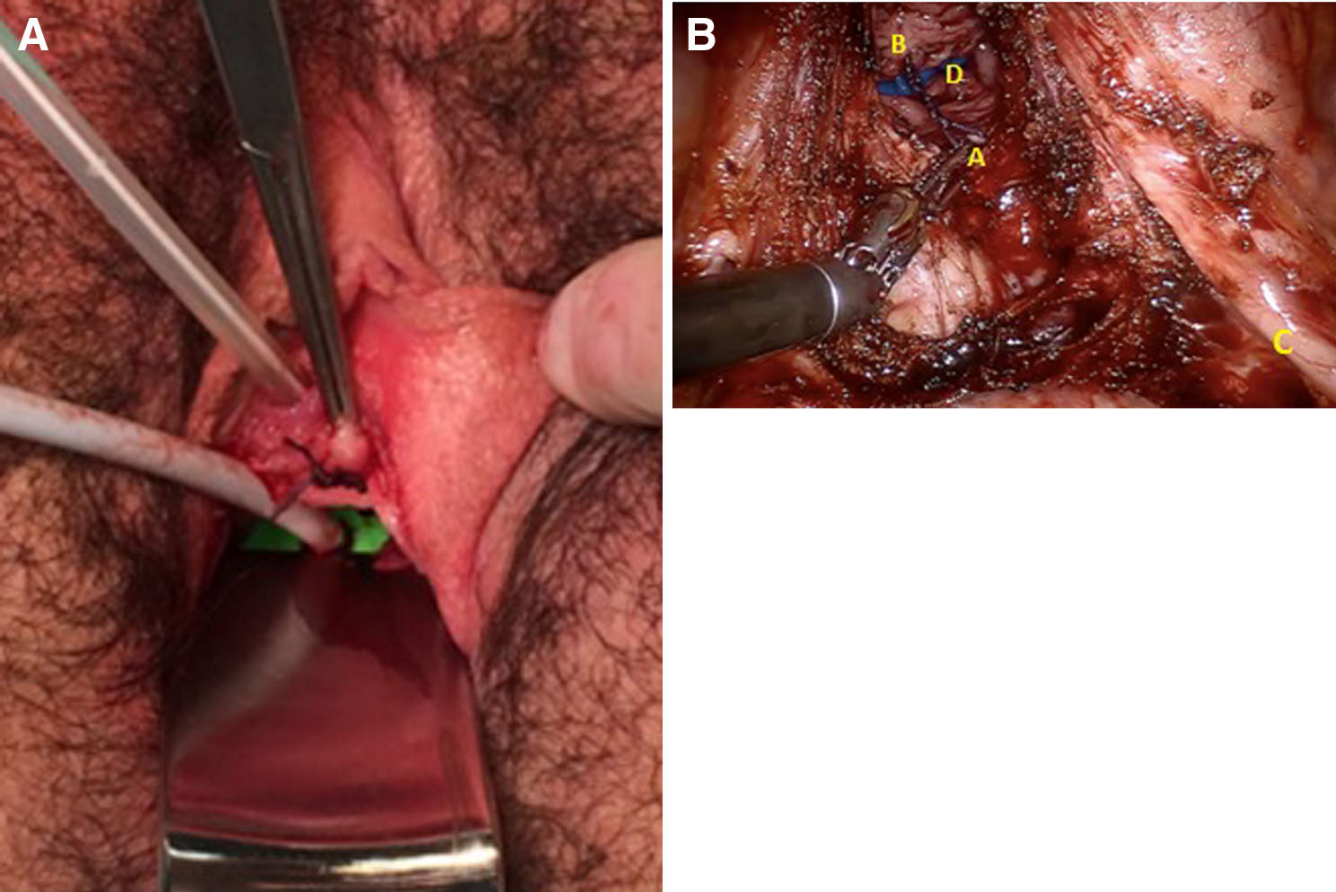
**A** Bordering landmark from the outside prior to colpectomy. Vaginal epithelium 15 mm around the ostium urethrae is necessary for the urethral anastomosis for further phalloplasty surgery. A suture needs to be placed prior to laparoscopic colpectomy to define this border. During the colpectomy, the suture is used a landmark as shown in (**B**). **B** Bordering landmark (*A*) from the inside during the colpectomy, vaginal epithelium (*B*), urether (*C*) and the *blue* glove-covered gauze (*D*) (Color figure online)

1. Vaginal part of the colpectomy:

With the introduction of the uterine mobilizer (V-Care^®^ Uterine Manipulator, ConMed, Utica, USA) vaginally, a suture is placed in the vaginal epithelium 10 mm proximal of the urethra as a bordering landmark for the colpectomy (see Fig. 1A, B). Vaginal epithelium 15 mm around the ostium urethrae is not removed as this tissue will be used during urethra elongation to create a second protective layer covering the urethral anastomosis. A rectum cannula is placed in order to identify the rectum during the colpectomy.

2. Positioning and trocar placement:

A small incision was made approximately 20 cm above the pubic bone for insertion of a Veress needle to create a pneumoperitoneum up to 20 mmHg. A total of 5 trocars were used (4 for the robot arms and 1 for the assistant). The da Vinci^®^ surgical system (Intuitive Surgical, Inc., Sunnyvale, CA, USA) was positioned to the patient’s right side, with a scrub nurse or assistant to the left side of the patient. The robotic instruments used were the monopolar scissors, bipolar fenestrated forceps and the grasping forceps. During surgery, pneumoperitoneum was kept at 8–10 mmHg. The patient was now placed in Trendelenburg position for optimal visualization of the operating area.

3. TLH and BSO:

A robot-assisted TLH and BSO are performed according to a standardized protocol [17, 18]. The ureters are lateralized and dissected up to the vesicoureteric junction in preparation of the colpectomy. The uterus and adnexa are removed through the vagina. Maintenance of pneumoperitoneum is accomplished by introducing a glove-covered gauze in the distal part of the vagina at the rim of the introitus. Hereafter, the robot-assisted colpectomy is performed.

4. Robot-assisted laparoscopic colpectomy (Video):

Anteriorly, the epithelium is dissected carefully with monopolar scissors to ensure the coagulation of the well-defined vasculature of the vaginal wall. Dissection is completed to approximately one centimeter proximal of the urethra (marked by the suture, see Fig. 1B) and posteriorly up to the level of the posterior commissure. The epithelium removed is as thin as possible to prevent nerve injury to adjacent structures, to prevent fistula to bladder, urethra or rectum, and to prevent bleeding from the perivaginal plexus. It is necessary to dissect the ureters up to the vesicoureteric junction in order to visualize the ureters during removal of proximal vaginal epithelium. After removal of the entire vaginal wall, the vaginal apex is sutured laparoscopically by suturing the remnants of the rectovaginal septum and endopelvic fascia of the vesicovaginal space together. During this step, it is crucial to carefully pay attention to the route of both ureters to avoid lesions or kinking. After hemostasis, the instruments are removed and the abdominal incisions closed. Finally, any residual epithelium at the level of the introitus is removed vaginally and the bulbocavernosus muscles are approximated with a few sutures to narrow the introitus. As a standard procedure, an absorbable hemostat is applied to the surgical field to prevent diffuse bleeding. The introitus is not closed, as access for the urethral anastomosis at a later stage is required.

### Postoperative care

Postoperative care was congruent with our standard vaginal colpectomy protocol consisting of bed rest during the first day after surgery and bladder training at postoperative day 2. Bladder training consisted of clamping the transurethral catheter twice for 3–4 h. When the urge to urinate is present both times, the catheter was removed. Spontaneous micturition must occur within 3–4 h. Postvoid residual urinary volume (PVR) was tested ultrasonographically after spontaneous micturition, and PVR must be <100 mL repeatedly (twice). Patients with a PVR of more than 100 mL were treated with an indwelling urinary catheter for 24 h. After successful bladder training and voiding, patients were discharged.

## Results

We performed robot-assisted TLH/BSO and RaCL in a total of 36 patients. Patient characteristics and results are shown in Table 1. All patients were nulliparous, and 30 of 36 patients (83.3%) were virginal. Three patients had previous minor abdominal surgery (appendectomy), and 33 had undergone a mastectomy. No conversions to laparotomy were encountered during RaLC.

**Table 1:** Patient characteristics

Number of patients	*N* = 36
Median age at surgery (years) (IQR)	23.5 (19.5–28.4)
Median BMI (kg/m^2^) (IQR)	22.2 (21–24.7)
Parity	36/36 nulliparous (100%)
Virgin	30/36 (83.3%)
Previous abdominal surgery	3/36 (8.3%)
Median bloodloss during surgery (mL) (IQR)	75 (30–200)
Median OR time (min) (IQR)	230 (197–278)
Median hospital stay (days) (IQR)	3 (2–3)
Conversion to laparotomy	0/36 (0%)
Major complications	1/36 (2.8%)
Postoperative bleeding with readmission	1/36 (2.8%)
Fistula (vesicovaginal or rectovaginal)	0/36 (0%)
Minor complications	8/36 (22%)
Urinary tract infection	2/36 (5.6%)
Urinary retention needing catheter	6/36 (16.7%)

Data are reported as median (interquartile range) or as *n* (percentage)

**Fig. 2:**
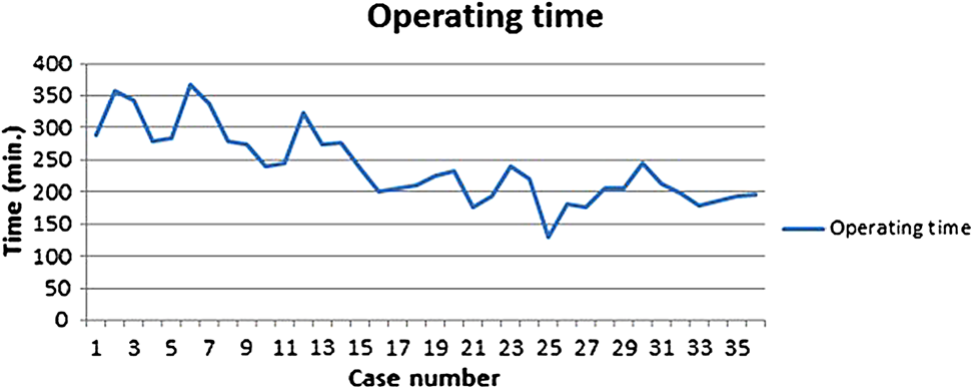
Learning curve for operating time in minutes

**Fig. 3:**
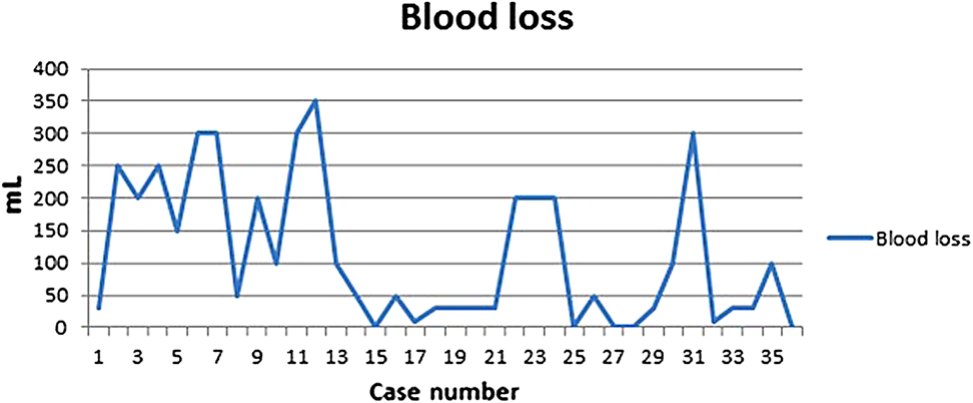
Learning curve for bloodloss in mL

Length of the procedure was acceptable with a median of 230 min (197–278), and median blood loss was 75 mL (30–200). Despite the fact that a very experienced robot surgeon executed all procedures, a clear learning curve can be observed in terms of intra-operative blood loss and gradual reduction in surgery time. Comparing the last 18 cases with the first 18 cases, median surgery time reduced from 278 to 197 min (*p* = 0.00) and median blood loss reduced from 175 to 30 ml (*p* = 0.01) (Figs. 2, 3).

We defined our complications as minor or major according to the study of Mourits et al. [19]. Some complications occurred: mainly minor and related to urinary voiding (see Table 1). One patient (2.8%) with a major complication (postoperative bleeding with readmission and transfusion) and 7 patients (19%) with minor complications were reported (2 urinary tract infection (UTI) and 6 urinary retention needing a catheter).

Median hospital stay was 3 (2–3) days postoperatively. At discharge, six patients experienced urine voiding problems requiring an indwelling catheter for a longer period of time (in accordance with standard urological protocols). After 72 h, PVR was tested again. In 5 patients, PVR was below 100 mL and the catheter was removed. Only one patient still had ongoing urine voiding problems after 72 h with bladder spasms due to a UTI. The urinary catheter was kept in place, and oral antibiotics were started. After an additional 48 h, the catheter was successfully removed.

Six out of eight patients had their catheters successfully removed at day 1 postoperatively after bladder training and sonographic confirmation of a PVR <100 mL.

Successful catheter removal continued on day 2 (13/17 patients) and day 3 (8/11 patients). The 8 patients, for whom the catheters were not successfully removed, had their catheter removed 48 h (2 patients), 72 h (6 patients) and 5 days after surgery (1 patient).

One patient, in the group of the first 18 operated patients, was readmitted 8 days after discharge because of vaginal bleeding. Bloodloss was most likely due to bleeding of the vaginal wall. At admission, blood loss was minimal and rectal palpation did not show a hematoma. Bleeding stopped spontaneously; however, 2 units of packed cells was administered due to significant hemoglobin drop (hemoglobin 7.7 mmol/L with hematocrit 0.36 L/L to hemoglobin 4.7 mmol/L with hematocrit 0.36 L/L). No patients had other serious adverse events or long-term sequelae.

## Discussion

### Main findings

Herein, we have established that RaLC is a feasible procedure in FtM transgender patients; a clear learning curve could be observed concerning surgery time and blood loss. In this series of patients, we have encountered one major postoperative complication (1 patient with postoperative bleeding) and 7 minor postoperative complications (5 patients with urine voiding problems, 1 patient with an UTI and 1 patient with urine voiding problems and an UTI). All 8 complications were successfully treated or resolved with conservative treatment.

### Strengths and limitations

Although this study has its limitations, such as a small number of patients and a relatively short follow-up period, the study we present shows the safety, feasibility and efficacy of the robot-assisted colpectomy combined with robot TLH/BSO as a single-step surgical procedure.

Consistent with the IDEAL criteria (Idea, Development, Exploration, Assessment and Long-term study) for the evaluation and implementation of an innovational surgical procedure, this is a prospective development study with an initial small group of patients, where a clear description of the surgery is outlined, without omissions and including learning curves as well. As these characteristics represent the stage 2a ‘Development’ based on these IDEAL criteria, this stage may be assigned to this study [20].

### Comparison to other data and clinical implications

In this study, we did not encounter severe complications such as fistula. However, the number of patients included in this pilot study is too low to conclude (yet) that RaLC is a safer procedure than vaginal colpectomy. Also there is very little literature available showing complication rates for vaginal colpectomy in patients.

Vaginal (partial) colpectomy is mainly described in patients with deep infiltrating endometriosis and in patients with cancer and may therefore have higher complication risk than our patient category. Complications described are mostly urological: 3.5–14.3% persistent urinary retention [12, 13, 21], and one paper describes 12.5% bladder perforation (1/8 patients) [22]. Complication rates for fistula (vesicovaginal or rectovaginal) are not reported in the literature, although there is a reported 0.7% incidence on rectal lacerations during surgery that involved the posterior vaginal wall [23]. In this series of patients, published by Hoffman et al. [23], the majority of procedures to the posterior vaginal wall included was for prolapse; other procedures were for neoplasia and transgender. In the 6 patients included, there was one serious adverse event: a rectal laceration of 2 cm (1 of 6 patients; 16.6% in this particular subgroup) [23]. None of these patients had further postoperative sequelae.

Sehnal et al. [24] published in 2008 a prospective randomized controlled trial in which they compared 3 methods of hysterectomy (through median laparotomy, Pfannenstiel incision or laparoscopy) in 61 FtM patients. The authors conclude that TLH is the best treatment because of better wound healing, low complication rate, short admission time and no damage to the rectus abdominis muscle and epigastric vessels (important for phalloplasty using a rectus abdominis flap). In none of the cases, a direct (vaginal) colpectomy was performed. Furthermore, they did not include vaginal hysterectomy as they deemed this procedure too difficult in this patient group. Kaiser et al. [25] published in 2011 a series of 106 FtM patients undergoing bilateral subcutaneous mastectomy, vaginal hysterectomy with BSO and complete vaginal colpectomy in the same surgical session. Although a series of more than 100 colpectomies in a transgender population is remarkable, Kaiser et al. did not describe any further detail regarding technique and results of colpectomy specifically. The conversion risk to laparotomy in this study was 2.8% of cases, and the major complication rate was 5.4% (bowel perforation 0.9%, bladder perforation 0.9%, compartment syndrome 1.8%, intra-abdominal bleeding 1.8%) [25.] If the vaginal approach is preferred and the narrow vagina limits appropriate access, one may consider the Schuchardt incision. This incision has been used for radical vaginal hysterectomy. Briefly, the skin is incised over 3–4 cm from the lower and middle third of the left labium majus. The vaginal wall, which is now under tension, is incised down to the levator ani muscle, and the rectum is displaced. The layers of the perineum are divided to expose the levator, which is then divided almost completely [26]. Schuchardt technique is known to cause considerable morbidity, such as an increased risk of bleeding, wound dehiscence, nerve damage, vaginal vault prolapse and in rare cases bowel evisceration. The preceding clearly shows that laparoscopic hysterectomy in patients is not inferior to vaginal hysterectomy.

Benefits of our technique over the vaginal route include an excellent exposure of the operating area with a complete overview of the vaginal epithelium, blood loss is minimal, postoperative recovery is fast with rapid postoperative mobilization and discharge, and there is almost immediate regain of bladder function after surgery. Admission to the hospital is longer after RaLC than after other laparoscopic procedures. This might be due to prudence with a new technique and unknown postoperative sequelae. This is underlined by the adjustment of the protocol concerning admission period during our cohort study. Further decrease in admission period is feasible. This is in line with the fact that more and more laparoscopic and robotic-assisted hysterectomies are executed in daycare [27–32].

Since patient recovery in our study was fast, we considered removing the catheter immediately after surgery instead of day 2. Joshi et al. [33] found no difference in UTI between patients who had their catheter removed immediately after an abdominal hysterectomy compared to patients who had their catheter removed after 24 h, while recatheterization was slightly more frequent in the early removal group. Although these patients are not completely comparable to colpectomy patients, it seems that early removal of urinary catheters in colpectomy patients (day 1) should be feasible.

The clinical implications are not yet clear but may include a faster return to work, less complications and less blood loss. Although these results are promising, this is a complex procedure even for the experienced robot surgeon and a clear learning curve is seen. Implementation of this technique on a larger scale and by more surgeons might not show the same positive results. The technique is still in the developmental phase according to the IDEAL criteria. Therefore, we advise to utilize this technique solely in a highly specialized center in a research setting in order to evaluate the technique on a larger scale.

To our knowledge, this is the first study to report on RaLC combined with robot TLH and BSO in FtM patients. Future research should be directed toward quality of life assessment and long-term follow-up. Furthermore, RaLC in combination with robot TLH/BSO needs to be compared to the standard two-step procedure of TLH/BSO and consecutive vaginal colpectomy. Also analysis should include complication rates of subsequent phalloplasty procedures.

## Electronic supplementary material

Below is the link to the electronic supplementary material.

Supplementary material 1 (MP4 305317 kb)
